# Time evaluation and its accuracy in eating disorders: differences in relation to interoceptive awareness

**DOI:** 10.1007/s40519-022-01394-7

**Published:** 2022-04-12

**Authors:** Paolo Meneguzzo, Cecilia Mancini, Aurora Ormitti, Elisa Bonello, Patrizia Todisco

**Affiliations:** 1 Present Address: Department of Neuroscience, University of Padova, Via Giustiniani 5, 35128 Padova, Italy; 2 Padova Neuroscience Center, University of Padova, Padova, Italy Via Giuseppe Orus, 2, 35131; 3 Eating Disorders Unit, Casa di Cura Villa Margherita, Via Costacolonna 6, Arcugnano, 36057 Vicenza, Italy; 4 Experimental Medicine Department, Food Science and Human Nutrition Research Unit, Sapienza University of Rome, Rome, Italy

**Keywords:** Eating disorders, Time estimation, Bodily awareness, Anorexia nervosa, Bulimia nervosa, Binge eating disorder

## Abstract

**Purpose:**

Time evaluation has been poorly studied in eating disorder (ED) patients despite its relationship with body awareness, which is a core psychopathological feature in EDs and is influenced by impulsivity, interoception, and working memory. This study aims to evaluate time estimation and its accuracy across the ED spectrum in connection with specific and general psychopathology.

**Methods:**

A group of 215 women was enrolled in a computerized task involving the estimation of 1-min intervals. Impulsivity and body awareness constructs (self-monitoring, depersonalization, interoceptive deficit) were evaluated and examined for significant correlations with time estimation and the accuracy of the measure.

**Results:**

Patients with EDs showed an impaired ability to estimate time, with an accuracy that positively correlated with compulsive self-monitoring (*p* = 0.03). Differences between diagnostic subgroups showed an overestimation of time in anorexia nervosa patients and an underestimation of time in binge eating disorder patients, whose time estimation was also less accurate.

**Conclusion:**

The relationship between time estimation and compulsive self- monitoring might corroborate the presence of an imbalanced integration of information in patients with EDs that was not present in the community women included in the study. Time perception should be further evaluated in the ED field, and longitudinal changes due to psychopathological recovery or BMI changes should be examined.

**Level of evidence:**

Level III: Evidence obtained from a well-designed cohort or case–control analytic study.

## Introduction

Eating disorders (EDs) are a severe group of psychiatric conditions characterized by the influence of body weight or shape on self-evaluation, a disturbance in the way one’s body or shape is experienced, dysfunctional eating behaviors, and difficulties in interoceptive awareness [1–3]. Recently, several authors have proposed interoception as a central feature of the development and maintenance of EDs [4], showing that higher levels of eating psychopathology are negatively associated with the core definition of interoception: body listening, trust, and the ability to tolerate bodily sensations [5, 6]. Interoception exhibits connections with body image [7] and emotional regulation [8], and, therefore, it plays a relevant role in the outcome of ED treatments [9]. Interoception also refers to the internal state of the body, its relationship with the external world, and a healthy relationship between the brain and body—all of which are impaired in patients with EDs [10]. Different methodologies have been applied in the evaluation of interoception in EDs, including self-report questionnaires and heartbeat, pain, and respiratory detection [11]. Neuroimaging data have already shown impaired connections of the somatosensory, limbic, and interoceptive networks in various ED subsamples [11–15]. The neurobiological alterations found in the insula and in the somatosensory networks have a deep connection with both the definition of self and the interoception abilities of patients [12, 15, 16], playing a possibly relevant role in the neuropsychological and behavioral domains of ED patients. This role requires further investigation.

Classically, interoception has been related to impulsivity traits [17] as a mediator system between cognitive control and the impulsive system [18], with physiological cues that have been considered behavioral guides of behaviors in specific risk-taking scenarios [19]. Recent literature has corroborated this evidence, integrating impulsivity and interoceptive multi-dimensional constructs and showing that interoceptive sensibility has contributed to emotional impulsivity, while interoceptive accuracy is related to non-planning impulsivity [20]. Interoception has also been associated with the experience and estimation of time in the general population, showing a blending between these two constructs [21]. The hypothesis that time perception and estimation arise from the accumulation of bodily information in the insula has become widely most accepted [22], even if additional variables are taken into consideration [23]. Recent neurophysiological evidence has confirmed the embodied nature of time estimation [21], showing that people with better interoceptive accuracy are more accurate in time estimation, with an overlap between interoception and time estimation in the activity of different brain structures like supplementary motor area and the striatum [24, 25].

In the ED literature, data are available for impulsivity traits [26, 27]. In addition, there is a growing interest in interoceptive abilities [6, 28], but little evidence about time estimation is available. Indeed, patients with EDs presented a low sensibility to the duration of food picture exposure, with patients with anorexia nervosa (AN) overestimating time [29]. In contrast, another study found that adolescent patients with AN underestimate time when asked to evaluate the duration of a specific probe on a screen, showing a decreased timing accuracy related to the delayed reward processes of patients [30]. No studies have evaluated time perception in patients with bulimia nervosa or binge eating disorder. To our knowledge, no other study has evaluated time estimation in ED patients. However, a growing body of literature has shown the presence of an impairment in the ability to perceive time in people under high levels of distress [31].

Thus, this study aims to investigate the perception of time in a large sample of patients with EDs, exploring the possible relationships between demographic data, impulsivity, and eating psychopathology. Based on data from the literature, we hypothesized that patients with EDs present an impaired ability to evaluate time compared to controls due to impaired body awareness and difficulties in interoceptive abilities. Our second hypothesis was that there would be differences between patients with ED and community women (CW) regarding time estimation, impulsive traits, and psychopathology.

## Methods

### Participants

When admitted for inpatient treatment at the Eating Disorder Unit of the Casa di Cura Villa Margherita (in Arcugnano—Vicenza, Italy), 172 female ED patients were enrolled in this study: 46 women with restrictive anorexia nervosa (ANr), 37 women with binge–purge AN (ANbp), 49 women with bulimia nervosa (BN), and 40 women with binge eating disorder (BED). All participants were between 16 and 50 years old and were evaluated by a trained psychiatrist through a structured interview. The diagnoses were formulated according to the criteria of the Diagnostic and Statistical Manual of Mental Disorders 5 [3]. The exclusion criteria were as follows: male gender; prior or current traumatic brain injury; or a lifetime history of any neurological, systemic, and/or severe psychiatric illness in comorbidity (suicidality, alcohol or substance use, psychotic features). For the comparison, a convenient group of 43 community women (CW) was enrolled in the study via a public call for volunteers in psychological experiments through social media pages. The exclusion criteria for CWs, who were recruited from the general population and evaluated by a trained psychiatrist, included a body mass index (BMI) below 18.5 kg/m^2^; a first-degree relative with a lifetime eating disorder; a prior or current traumatic brain injury; any neurological, psychiatric, or systemic illness; and the use of psychoactive medication.

All participants gave written informed consent during their recruitment. If a participant was under 18 years old, consent was collected from their parents. The study protocol was in accordance with the ethical standards of the Declaration of Helsinki and its later amendments and was approved by the Vicenza Ethics Committee (47/21).

### Procedure and the time evaluation task

During the first week of inpatient treatment, recruited patients filled out a series of psychological questionnaires. Afterward, they were asked to perform the time evaluation (TE) task. The task was performed in the morning, in a quiet room without any clock or watch present. After examining the current literature, we decided to use a one-minute detection task (60,000 ms). This decision was supported by the literature on neurodevelopmental disorders, which has shown more specific brain activity linked to long-time estimation, obtaining results that were less influence by general brain processes, such as attentional resources or the encoding and retrieval of information [32, 33]. The TE task consisted of a computerized test in which participants were instructed to start and stop their estimations five times by pressing the space bar. A black screen was presented during the task, and an instruction page appeared after the stop of the chronometer, explaining to the participants how many trails they had already completed out of five. Between each block, participants could stop and rest. The TE outcomes consisted of five time measures—one for each block—that were used to evaluate the participants’ time estimations and accuracy on both the first block and on the entire task. Both outcomes were collected to allow differentiation between under and over estimation of time, and the ability to measure time correctly. The task was performed with OpenSesame (https://osdoc.cogsci.nl/) on a 17″ laptop (2-GHz processor with 8 GB of RAM).

### Self-reported measures

Different psychopathological facets were evaluated for this study using validated self-report questionnaires. Specific eating psychopathology was evaluated with the Eating Disorder Examination Questionnaire (EDE-Q), which consisted of 28 self-report items regarding specific aspects of eating disorder psychopathology [34]. Different facets of interoceptive awareness were evaluated using the interoceptive deficits (ID) specific subscale of the Eating Disorders Inventory-3 (EDI-3). This scale is composed of a total of nine self-report items focused on internal states and their acceptance [35] and two subscales of the body uneasiness test (BUT) for a total of 11 self-report items. The two included subscales of the BUT are the compulsive self-monitoring subscale (CSM), which investigates the compulsive checking of physical appearance, and the depersonalization subscale (D), which investigates detached and estranged feelings toward the body [36]. Finally, impulsivity was evaluated with the UPPS-P Impulsive Behavior Scale, a 20-item self-report scale with five specific subscales: positive urgency, negative urgency, lack of premeditation, lack of perseverance, and sensation seeking [37].

### Statistical analyses

Demographic characteristics among the different groups were evaluated using an ANOVA analysis post hoc, with the Bonferroni correction used for the pairwise comparison. Results were confirmed with an ANCOVA analysis that used BMI as a covariate due to the presence of significant differences among the groups. Effect sizes were evaluated with ε^2^ due to the non-parametric distribution (small: 0.02 ≤ ε^2^ < 0.13; medium: 0.13 ≤ ε^2^ < 0.26; large: ε^2^ ≥ 0.26). The TE task was composed of five separate measurements (recorded in ms), and an average time of response was calculated for each participant. The distribution of the TE task results among participants was evaluated using the Kruskal–Wallis test, with the Bonferroni correction used for the pairwise comparisons due to the non-parametric distribution of the values. Accuracy was evaluated as follows: 1—| (60.000—time evaluated) / 60.000 |, with values from 0 (low accuracy) to 1 (high accuracy), both for the first minute (T1) and for the average time estimated (TA), looking to elicit the influence of attention on the task performances [38]. Differences in accuracy were evaluated with the Kruskal–Wallis test and pairwise post hoc analyses. Given the sample size, an ANCOVA with BMI as a covariate was performed to confirm the results. Relationships between accuracy and different psychological scores were evaluated with Spearman’s ρ due to the non-parametric distribution and with Benjamini–Hochberg correction to avoid type 1 error. The p values were considered significant at 0.05, and all the analyses were performed with SPSS software 25.0.

## Results

Demographic characteristics of the participants are listed in Table 1. Significant differences reported in Table 1 between groups were confirmed with BMI as a covariate, except for negative impulsivity (*p* = 0.289) and lack of premeditation (*p* = 0.155). While none of the CW participants were receiving any drug treatments, 140 out of 172 patients (82%) were receiving drug treatments with selective serotonin reuptake inhibitors or second-generation antipsychotic. There were no differences between diagnostic subgroups distribution of drug treatments.

**Table 1:** Demographic characteristics of the sample

	ANr *n* = 46	ANbp *n* = 37	BN *n* = 49	BED *n* = 40	CW *n* = 43	F (*p*) ε^2^	Post hoc
Age, years	24.54 (8.99)	25.78 (9.08)	25.37 (8.74)	29.79 (11.22)	24.60 (5.65)	2.217 (.068) .017	
BMI, kg/m^2^	14.82 (1.92)	16.21 (1.68)	22.69 (6.72)	35.76 (5.96)	21.50 (2.74)	130.764 (< .001) .841	ANr < BN (*p* < .001) ANr < BED (*p* < .001) ANr < CW (*p* < .001) ANbp < BN (*p* < .001) ANbp < BED (*p* < .001) ANbp < CW (*p* < .001) BN < BED (*p* < .001) CW < BED (*p* < .001)
EDE-Q Global	3.28 (1.30)	4.23 (1.38)	4.32 (1.22)	3.90 (1.26)	1.50 (1.70)	28.376 (< .001) .326	CW < ANr (*p* < .001) CW < ANbp (*p* < .001) CW < BN (*p* < .001) CW < BED (*p* < .001)
EDI-3 ID	16.53 (8.63)	22.06 (7.38)	20.48 (7.94)	18.59 (7.38)	3.28 (1.92)	47.312 (< .001) .491	ANr < ANbp (*p* = .007) CW < ANr (*p* < .001) CW < ANbp (*p* < .001) CW < BN (*p* < .001) CW < BED (*p* < .001)
UPPS-P							
NU	9.93 (3.84)	9.60 (2.73)	8.29 (3.03)	8.50 (2.37)	9.23 (1.77)	2.482 (.045) .044	
PU	12.14 (3.56)	10.60 (2.28)	9.80 (3.31)	9.62 (2.87)	8.47 (2.86)	8.498 (< .001) .167	BN < ANr (*p* = .004) BED < ANr (*p* = .004) CW < ANr (*p* < .001)
LPre	7.02 (3.20)	8.00 (3.24)	8.22 (2.54)	9.21 (2.95)	7.23 (2.42)	3.499 (.009) .085	
LPer	7.33 (3.53)	8.83 (3.78)	8.24 (3.10)	10.09 (3.21)	7.56 (3.22)	4.042 (.004) .083	ANr < BED (*p* = .004)
SS	12.00 (3.98)	10.80 (2.99)	10.18 (3.21)	10.26 (3.46)	9.14 (3.45)	4.153 (.003) .087	CW < ANr (*p* = .001)
BUT							
CSM	2.49 (1.41)	2.79 (1.25)	2.99 (1.28)	1.31 (0.81)	0.96 (0.90)	18.167 (< .001) .301	BED < ANr (*p* < .001) CW < ANr (*p* < .001) BED < ANbp (*p* < .001) CW < ANbp (*p* < .001) BED < BN (*p* < .001) CW < BN (*p* < .001)
D	1.70 (1.00)	2.13 (1.10)	2.28 (1.12)	1.43 (1.15)	0.44 (0.62)	13.140 (< .001) .236	CW < ANr (*p* < .001) CW < ANbp (*p* < .001) CW < BN (*p* < .001) CW < BED (*p* = .008) BED < BN (*p* = .004)

*AN* anorexia nervosa, *BN* bulimia nervosa, *BED* binge eating disorder, *CW* community women, *r* restrictive, *bp* bing–-purge, *BMI* body mass index, *EDE-Q* Eating Disorder Examination Questionnaire, *EDI-3 ID* Eating Disorder Inventory-3 interoceptive deficit subscale, *UPPS-P Impulsive Behavior Scale-NU* negative urgency, *PU* positive urgency, *LPre* lack of premeditation, *LPer* lack of perseverance, *SS* sensation seeking; BUT: body uneasiness test, *D* depersonalization, CSM: compulsive self-monitoring. Post hoc analysis with Bonferroni correction

### Time perception

No difference in the ability to estimate time emerged when comparing the patients with EDs and the CWs (T1, ED 64,077 ± 20,840 ms, *Z* = -1.248, *p* = 0.212; TA, 67,281 ± 23,979, *Z* = − 0.594, *p* = 0.552). However, ED patients showed less accuracy in the estimation of 1 min than did CWs (ED: 0.73 ± 0.25; *Z* = − 2.979, *p* = 0.003), but no difference emerged for the mean accuracy of the five tests (ED: 0.73 ± 0.32; *Z* = − 1.542, *p* = 0.123).

Comparing time perception between subgroups, both the AN subgroups showed longer estimation times, with an average elongated time that was significantly different from both BED patients and CWs. Patients with BED reported underestimations of time compared to CW and had the lowest accuracy rates. These results were confirmed with BMI as a covariate. See Table 2 for details.

**Table 2:** Time estimation and accuracy

	ANr *n* = 46	ANbp *n* = 37	BN *n* = 49	BED *n* = 40	CW *n* = 43	H (*p*) ε^2^	Post hoc
T1, ms	69,305 (21,019)	72,769 (22,998)	62,147 (14,770)	50,330 (18,753)	61,200 (11,137)	25.820 (< .001) .120	BED < ANr (< .001) BED < ANbp (< .001) CW < ANr (.010)
T2, ms	70,298 (24,287)	72,124 (31,709)	63,852 (22,657)	54,632 (18,911)	62,312 (12,749)	12.819 (.012) .067	BED < ANr (.001)
T3, ms	70,413 (27,884)	72,391 (29,874)	63,250 (14,856)	58,449 (21,151)	63,516 (12,879)	4.904 (.297) .024	
T4, ms	70,941 (25,230)	73,500 (28,886)	63,805 (13,368)	58,765 (20,982)	62,822 (12,943)	9.814 (.044) .052	
T5, ms	67,649 (21,126)	73,227 (27,071)	60,201 (13,815)	62,872 (29,032)	63,933 (13,447)	8.314 (.081) .041	
TA, ms	71,712 (25,282)	72,802 (27,033)	62,447 (13,378)	61,951 (28,694)	62,850 (11,540)	9.770 (.044) .050	
Accuracy T1	0.71 (0.25)	0.72 (0.34)	0.82 (0.17)	0.65 (0.22)	0.85 (0.12)	24.989 (< .001) .120	BED < ANbp (.010) BED < BN (< .001) BED < CW (< .001) ANr < CW (.004)
Accuracy TA	0.68 (0.34)	0.73 (0.42)	0.84 (0.16)	0.65 (0.33)	0.84 (0.12)	14.443 (.006) .069	BED < CW (.010) BED < BN (.002) ANr < BN (.006)

*AN* anorexia nervosa, *BN* bulimia nervosa, *BED* binge eating disorder, *CW* community women, *r* restrictive, *bp* bing–-purge, *T* time estimation (in ms) in the different trials, *TA* time average

### Correlation analyses

Different associations between time estimation and psychological constructs emerged between patients with EDs and CW. Regarding T1 and TA, participants with EDs showed a positive correlation between TA and negative urgency (*p* = 0.034) and negative correlations with age and BMI. Eating disorder patients also showed a positive correlation between accuracy TA and compulsive self-monitoring (*p* = 0.030). See Table 3 for details and for correlations between other constructs included in the analysis. No specific correlations emerged for CW regarding time estimation or accuracy and the evaluated psychological constructs. See Table 4 for details.

**Table 3:** Spearman’s correlation analysis in patients with EDs

		1	2	3	4	5	6	7	8	9	10	11	12	13	14
1	Age	–													
2	BMI	0.07	–												
3	T1	− 0.22*	− 0.33***	–											
4	TA	− 0.28**	− 0.18	0.79***	-										
5	Accuracy T1	0.06	− 0.06	− 0.20*	− 0.22	-									
6	Accuracy TA	0.03	0.01	− 0.23*	− 0.36***	0.71***	–								
7	EDE-Q Global	− 0.05	0.01	− 0.03	− 0.08	− 0.04	− 0.14	–							
8	ID	− 0.21	− 0.01	− 0.09	− 0.08	0.07	0.13	0.36***	–						
9	NU	.02	− 0.21	.11	.20*	.05	− 0.03	.01	− 0.32***	–					
10	PU	0.12	− 0.30***	0.08	0.05	− 0.01	.02	− 0.15	− 0.31***	0.50***	–				
11	LPre	− 0.02	0.24**	− 0.11	− 0.09	− 0.18	− 0.14	0.13	0.20*	− 0.26**	− 0.30***	–			
12	LPer	− 0.03	0.20*	− 0.13	− 0.08	− 0.03	− 0.05	0.05	0.13	− 0.20*	− 0.13	0.56***	–		
13	SS	0.37***	− 0.15	− 0.03	− 0.06	− 0.05	− 0.12	.01	− 0.27**	.22	.51***	− 0.10	.02	–	
14	CSM	− 0.32***	− 0.22*	0.16	0.05	0.17	0.21*	0.49***	-40***	− 0.07	− 0.01	− 0.10	− 0.03	− 0.07	–
15	D	− 0.10	− 0.04	0.05	0.01	0.11	0.03	0.42***	.33***	− 0.06	− 0.06	− 0.05	0.02	− 0.03	0.50***

*BMI* body mass index, *EDE-Q* Eating Disorder Examination Questionnaire, *ID* interoceptive deficit subscale, *NU* negative urgency, *PU* positive urgency, *LPre* lack of premeditation, *LPer* lack of perseverance, *SS* sensation seeking, *D* depersonalization, *CSM* compulsive self-monitoring

^*^p < 0.05; **p < 0.01; ***p < 0.001, with Benjamini–Hochberg correction

**Table 4:** Spearman’s correlation analysis in CW

		1	2	3	4	5	6	7	8	9	10	11	12	13	14
1	Age	–													
2	BMI	0.04	–												
3	T1	0.09	0.17	–											
4	TA	0.18	0.25	0.87***	–										
5	Accuracy T1	.11	− 0.08	− 0.11	− 0.10	–									
6	Accuracy TA	0.07	− 0.24	− 0.21	− 0.31	0.66***	–								
7	EDE-Q Global	− 0.15	0.13	− 0.09	0.02	− 0.05	− 0.01	–							
8	ID	0.03	0.07	− 0.13	− 0.06	− 0.29	− 0.18	0.33	–						
9	NU	− 0.24	0.28	− 0.05	− 0.04	0.09	0.09	0.35	0.22	–					
10	PU	.07	.32	− 0.18	− 0.20	0.22	0.25	0.39	0.07	0.63***	–				
11	LPre	− 0.01	0.30	0.09	0.15	0.23	0.33	0.34	− 0.15	0.43*	0.59**	–			
12	LPer	− 0.24	− 0.10	− 0.01	− 0.05	0.05	0.17	0.19	0.01	0.37	0.18	0.49*	–		
13	SS	− 0.16	0.16	− 0.29	− 0.26	0.08	− 0.01	0.16	0.08	0.16	0.45*	0.16	− 0.05	–	
14	CSM	− 0.30	− 0.03	− 0.25	− 0.34	− 0.41	− 0.21	0.24	0.61	0.07	0.08	− 0.12	− 0.08	0.23	–
15	D	− 0.44	0.06	0.10	0.06	− 0.54	− 0.48	− 0.12	0.21	− 0.05	− 0.07	− 0.14	− 0.04	0.26	0.70**

*BMI* body mass index, *EDE-Q* Eating Disorder Examination Questionnaire, *ID* interoceptive deficit subscale, *NU* negative urgency, *PU* positive urgency, *LPre* lack of premeditation, *LPer* lack of perseverance, *SS* sensation seeking, *D* depersonalization, *CSM* compulsive self-monitoring

^*^p < 0.05; **p < 0.01; ***p < 0.001, with Benjamini–Hochberg correction

## Discussion

This study aimed to evaluate preliminary evidence regarding the ability of patients with EDs to estimate 1 min, which could be tied to possible connections with specific psychopathological features and show an association with compulsive self-monitoring.

The major finding of this study is the presence of an impaired accuracy in ED patients regarding their ability to estimate time, with different specific profiles across the ED spectrum. This result only partially confirmed our main hypothesis. Indeed, both AN and BED patients presented a different accuracy level than CWs, while patients with BN did not. Time estimation has been connected to subjective orientation into autobiographical memories [39], and preliminary data have shown that ED patients have overgeneralized self-memories [40], showing a disconnection with personal time estimation. From this perspective, our results corroborate the idea of impaired self-orientation in ED patients, revealing a specific cognitive impairment that should be further investigated. This interoceptive imbalance could explain the slower evaluation of time in patients with AN and the behavioral impulsivity shown by patients with BED.

Looking at the correlation analyses, accuracy in time perception was negatively associated in our results with compulsive self-monitoring in patients with EDs. Compulsive self-monitoring is a psychological feature that has already been correlated with body image issues and emotional concerns, in both ED patients and the general population [41], and which might have a role in the impaired integration of bodily information. Body awareness has been related to self-monitoring during development [42], showing that a balanced integration of internal and external information is crucial for regular development and that this could influence the internal clock. In ED patients, self-monitoring is an essential aspect linked to the psychopathological core of the body and weight dissatisfaction [36], but it has also been related to parental bonding during development [43]. Trust in bodily sensations has already been underlined as a core element in AN [6, 28], and impairment in the integration of interoceptive information might have a role in the food-related implicit bias that has been found in patients with EDs [44]. From this perspective, a more robust knowledge about the integration of interoceptive information is needed, especially in patients with diagnoses other than AN. Interoceptive difficulties in ED patients have been proposed as an endophenotype [4] and the result of high levels of bodily and food-related anxiety [9, 45]. However, longitudinal studies are lacking. Our data failed to find associations with the others interoceptive constructs evaluated in our study. This could be linked to the multidimensional nature of interoception, which might be more correctly evaluated with a specific questionnaire like the multidimensional assessment of interoceptive awareness [5, 6]. This should be considered in future studies.

Another interesting result was the negative correlation between BMI and the accuracy of time estimations presented in the clinical sample. This result was also found in a study about patients with high weight [46], showing a possible link between dysfunctional weight behaviors and time estimation across the weight spectrum in the clinical population with dysfunctional eating behaviors. Body weight and eating concerns could be linked to impairment of the internal clock, which is linked to cognitive control, bodily awareness, and impulsivity [18], possibly also in regulating eating behaviors [46, 47].

Looking at impulsivity facets, negative urgency (i.e., the tendency to surrender to impulses when accompanied by negative emotions) showed a positive correlation with TA and a negative association with interoceptive difficulties in patients. These results corroborated the evidence about the connections among interoceptive awareness and emotional impulsivity [48, 49], underlining the need for a more comprehensive evaluation of impulsivity, bodily information integration, and cognitive control in patients with eating disorders, according to the tripartite model of interoception [18].

From a methodological perspective, this study applied a long-time estimation for the first time in the ED field. A previous study demonstrated that emotions and task duration influenced estimations of passing time [50], displaying a linear increase in the time estimated with the prolongment of the task. Moreover, the internal clock is usually linked to both internal and external information, revealing that estimation abilities could be influenced by different emotions and time duration [50]. However, this evidence is still preliminary. Therefore, future studies in the ED field should include different durations of the task and longitudinal evaluations, which would define the roles of various characteristics (e.g., BMI, specific and general psychopathology) on time perception.

## Strengths and limitations

Although this study included a large clinical population with different behavioral profiles, several limitations should be considered when examining the results. Firstly, the cross-sectional nature of this study did not permit the inference of causal relationships between interoception and EDs. Second, the self-report nature of the psychological evaluations may have influenced the results due to the clinical severity of the included patients. Indeed, AN patients reported high levels of impulsivity, and BED scores seem to be comparable to CWs. This could be linked to different facets of the reward system [51], which could be more compromised in severe patients like those included in this study. Third, we included only female participants; this impacts the possible generalization of the results, but it also increases the robustness of our findings by eliminating gender differences in time estimation abilities [52]. Fourth, our clinical sample included patients undergoing drug treatments that might have affected performance on the time task, and this aspect should be controlled for future studies including drug-naïve participants. Finally, future studies should include multidimensional questionnaires about interoception to allow differentiation between interoceptive facets.

## Conclusion

Despite the limitations of this study, we evaluated—for the first time—the time estimation abilities of a large sample of ED patients, revealing the presence of an impairment in their time estimation and accuracy compared to healthy peers. Specific correlations were found between time estimation difficulties and BMI and between time estimation difficulties and compulsive self-monitoring, suggesting the presence of an imbalanced integration of bodily information that impacted participants’ internal clocks. Both transdiagnostic features and specific diagnostic characteristics were elicited in our sample, proving that time estimation and the accuracy in its measurement should be considered separately in the ED spectrum. Specific impulsivity traits should also be considered due to the increasing data suggesting their roles in ED psychopathology. Future research should include tasks of various lengths of time and investigate time perception along the path to recovery, as well as targeting differences in clinical subgroups.

### What is already known on this subject?

Limited literature is available about time estimation in individuals with EDs. However, data available suggest impairment of their ability to correctly evaluate passing time.

### What this study adds?

For the first time in the literature, this study evaluated the ability and the accuracy of time estimation in patients on the ED spectrum, showing different results and relationships with body awareness. A possible association with impulsivity was also evaluated.

## Data Availability

The data that support the findings of this study are available from the corresponding author upon reasonable request.
